# The pleural fluid lactate dehydrogenase/adenosine deaminase ratio differentiates between tuberculous and parapneumonic pleural effusions

**DOI:** 10.1186/s12890-017-0526-z

**Published:** 2017-12-04

**Authors:** Jinlin Wang, Jun Liu, Xiaohong Xie, Panxiao Shen, Jianxing He, Yunxiang Zeng

**Affiliations:** 1 Department of Respiratory Disease, Guangzhou, China; 2 Department of Cardiothoracic Surgery, Guangzhou, China; 3 The State Key Laboratory of Respiratory Disease, China Clinical Research Centre for Respiratory Disease, Guangzhou Institute of Respiratory Disease, First Affiliated Hospital of Guangzhou Medical University, 151 Yanjiang Road, Guangzhou, Guangdong Province, 510120 China

**Keywords:** Pleural fluid, Lactate dehydrogenase, Adenosine deaminase, Tuberculous pleural effusion, Parapneumonic pleural effusion

## Abstract

**Background:**

Although pleural fluid lactate dehydrogenase (LDH) and adenosine deaminase (ADA) levels are often used to distinguish between tuberculous pleural effusion (TPE) and parapneumonic pleural effusion (PPE), this can be challenging as the LDH level may vary from normal to severely increased in PPE and a significantly elevated ADA is frequently measured in both conditions. In this study, we evaluated use of the pleural fluid LDH/ADA ratio as a new parameter to discriminate TPE from PPE.

**Methods:**

A retrospective study was conducted in patients with pathologically-confirmed TPE (*n* = 72) and PPE (*n* = 47) to compare pleural fluid LDH and ADA levels and LDH/ADA ratios between the 2 groups. A receiver operating characteristic (ROC) curve was constructed for identifying TPE.

**Results:**

The median pleural fluid LDH and ADA levels and LDH/ADA ratios in the TPE and PPE groups were: 364.5 U/L vs 4037 U/L (*P* < .001), 33.5 U/L vs 43.3 U/L (*P* = .249), and 10.88 vs 66.91 (*P* < .0001), respectively. An area under the ROC curve of 0.9663 was obtained using the LDH/ADA ratio as the indicator for TPE identification, and the sensitivity, specificity, positive likelihood ratio (PLR), and negative likelihood ratio (NLR) were, respectively, 93.62%, 93.06%, 13.48, and 0.068 at a cut-off level of 16.20.

**Conclusions:**

The pleural fluid LDH/ADA ratio, which can be determined from routine biochemical analysis, is highly predictive of TPE at a cut-off level of 16.20. Measurement of this parameter may be helpful for clinicians in distinguishing between TPE and PPE.

**Electronic supplementary material:**

The online version of this article (10.1186/s12890-017-0526-z) contains supplementary material, which is available to authorized users.

## Background

Pleural effusion, which is a commonly observed clinical manifestation, is associated with more than 50 recognized diseases and disorders. In most parts of world, subtypes of exudative effusions often seen in clinical practice include tuberculous pleural effusion (TPE), parapneumonic effusion (PPE), and malignant pleural effusion (MPE) [1, 2]. It is crucially important to differentiate TPE and PPE, which are curable conditions, from MPE, as misdiagnosis and delayed treatment can result in significant mortality and morbidity [3, 4]. A recent study found that only 31% of patients with TPE have a positive microbiological test result [5]. In contrast, an increased pleural fluid adenosine deaminase (ADA) level is frequently seen in TPE, which usually helps to discriminate it from PPE [6, 7]. Data from a meta-analysis [6] revealed a sensitivity and specificity as high as 92% and 90%, respectively, for the use of ADA in the diagnosis of TPE. However, a similar or even higher ADA level has occasionally been reported in PPE, particularly in patients with empyema [8–10].

**Table 1:** Comparison of clinical and laboratory findings between patients with TPE and PPE

Parameter	TPE (*n* = 72)	PPE (*n* = 47)	*P* Value
Demographic data:			
Age, years	51.5 (17–82)	59.0 (26–93)	.014
Male sex	49 (68.1%)	33 (75%)	
Blood values:			
Albumin, g/dl	67.25 (55.3–81.6)	66.1 (52.5–79.5)	.633
LDH, U/L	81.0 (111–321)	180.3 (118–433)	.618
Pleural fluid values:			
Protein, g/dl	50.0 (9.6–65.1)	47.6 (19.1–65)	.045
LDH, U/L	364.5 (55–1154)	4037 (103–48,730)	< .001
ADA, U/L	33.5 (4.5–75.9)	43.3 (2.0–344.1)	.249
LDH/ADA ratio	10.88 (3.65–21.81)	66.91 (9.04–411.4)	< .0001

Continuous variables are presented as the median (range) and qualitative variables as the number and percentage.

ADA, adenosine deaminase; LDH, lactate dehydrogenase; PPE, parapneumonic pleural effusion; TPE, tuberculous pleural effusion

**Table 2:** Comparison of pleural fluid LDH, ADA, and LDH/ADA ratio values between patients with TPE, UPPE, CPPE, and empyema

Parameter	TPE [A] (*n* = 72)	UPPE [B] (*n* = 23)	CPPE [C] (*n* = 15)	Empyema [D] (*n* = 9)	*P* Values		
					A vs B	A vs C	A vs D
Pleural LDH (U/L)	364.5 (55–1154)	316.8 (103–852)	2275 (1012–4446)	16,479 (1919–48,730)	.291	< .0001	< .0001
Pleural ADA (U/L)	33.5 (4.5–75.9)	10.55 (2.0–26.1)	37.9 (14.3–68.6)	137.9 (31.5–344.1)	< .0001	.377	< .0001
Pleural LDH/ADA ratio	10.88 (3.65–21.81)	41.33 (9.04–162.4)	67.3 (41.96–260.9)	87.50 (15.11–411.4)	< .0001	< .0001	< .0001

Values are medians (range)

ADA, adenosine deaminase; CPPE, complicated parapneumonic effusions; LDH, lactate dehydrogenase; PPE, parapneumonic pleural effusion; TPE, tuberculous pleural effusion UPPE; uncomplicated parapneumonic effusion

Management of pleural effusion is usually initiated after determining its transudative or exudative nature and comparing pleural fluid lactate dehydrogenase (LDH) and serum LDH levels according to Light’s criteria [11]. Clinical practice guidelines endorse the use of pleural fluid LDH and glucose to assist in the classification of patients with complicated parapneumonic effusions (CPPE) [12, 13]. However, an elevated pleural fluid LDH may present in TPE, PPE, and MPE, and the level is likely to range greatly from normal to extremely increased, which limits the use of LDH for identifying PPE in an individual patient due to the low sensitivity [9, 13, 14]. Therefore, it remains a challenge for clinicians to distinguish between patients with TPE and PPE from elevated pleural fluid ADA and LDH levels. As different mechanisms contribute to the elevation of ADA and LDH, it may be helpful to differentiate TPE from PPE by examining the pleural fluid LDH/ADA ratio, which has not been investigated in previous studies.

The aim of the present study was to evaluate the use of the pleural fluid LDH/ADA ratio as a new parameter to discriminate between TPE and PPE.

## Methods

### Study design and setting

A retrospective, non-randomized study of patients with confirmed diagnoses of TPE and PPE was conducted at a dedicated respiratory center (State Key Laboratory of Respiratory Disease and China Clinical Research Centre of Respiratory Disease, Guangzhou Institute of Respiratory Disease, Guangzhou).

### Patients

The available data for a total of 72 patients with TPE and 47 with PPE who were treated at our respiratory center between January 2014 and December 2015 were reviewed retrospectively. The inclusion criteria for patients with TPE were: (1) chronic granulomatous inflammation in pleural tissue; (2) a clinical response to anti-tuberculosis treatment; and (3) no pleural effusion or only a small amount observed in chest ultrasound examinations over the last 12 months. The criteria for inclusion of patients with PPE were: (1) exudative effusions associated with bacterial pneumonia, lung abscesses, or bronchiectasis; (2) absence of *Mycobacterium tuberculosis* (MTB) in pleural fluid obtained from serial thoracentesis procedures; (3) pathological manifestations of inflammatory pleuritis, pleural fibrosis and plaques, or chronic empyema, without evidence of MTB; and (4) remission and recovery for at least 3 months at follow-up visits after antibiotic treatment. Uncomplicated parapneumonic effusion (UPPE) was defined when patients responded to antibiotic treatment alone; complicated parapneumonic effusion (CPPE) was defined when nonpurulent-appearing effusions required medical interventions such as drainage and other procedures; and empyema was defined when there was frank pus in the pleural space [15].

The demographic and clinical data of the patients were collected for analysis of the pleural fluid LDH and ADA levels and the LDH/ADA ratio.

### Statistical analysis

Continuous variables were presented as the median and range, and qualitative variables were presented as the number and percentage. Intergroup differences were analysed statistically using SPSS® 17.0 (SPSS Inc., Chicago, IL, USA). Receiver operating characteristic (ROC) curves were used to identify the optimal cut-off points. The Wilcoxon signed-rank test or Fisher’s exact test were used for comparisons of the test results. Significance for statistical analyses was set at *P* < .05.

## Results

Data for a total of 72 patients with TPE and 47 with PPE were reviewed (Additional file 1: Excel). The data for the TPE patients revealed 4 who had positive MTB cultures (3 in pleural fluid, 2 in sputum), 4 with pleural fluid TB-DNA, and 1 who was positive on acid-fast bacillus (AFB) testing. Among the PPE patients, bacterial pathogens were identified in 10 cases (3 in pleural fluid, 2 in sputum, and 1 in blood 1), including streptococci in 4 patients and staphylococci in 3. In addition, 23 UPPE, 15 CPPE, and 9 empyema cases were respectively identified (according to the definitions specified above).

**Fig. 1:**
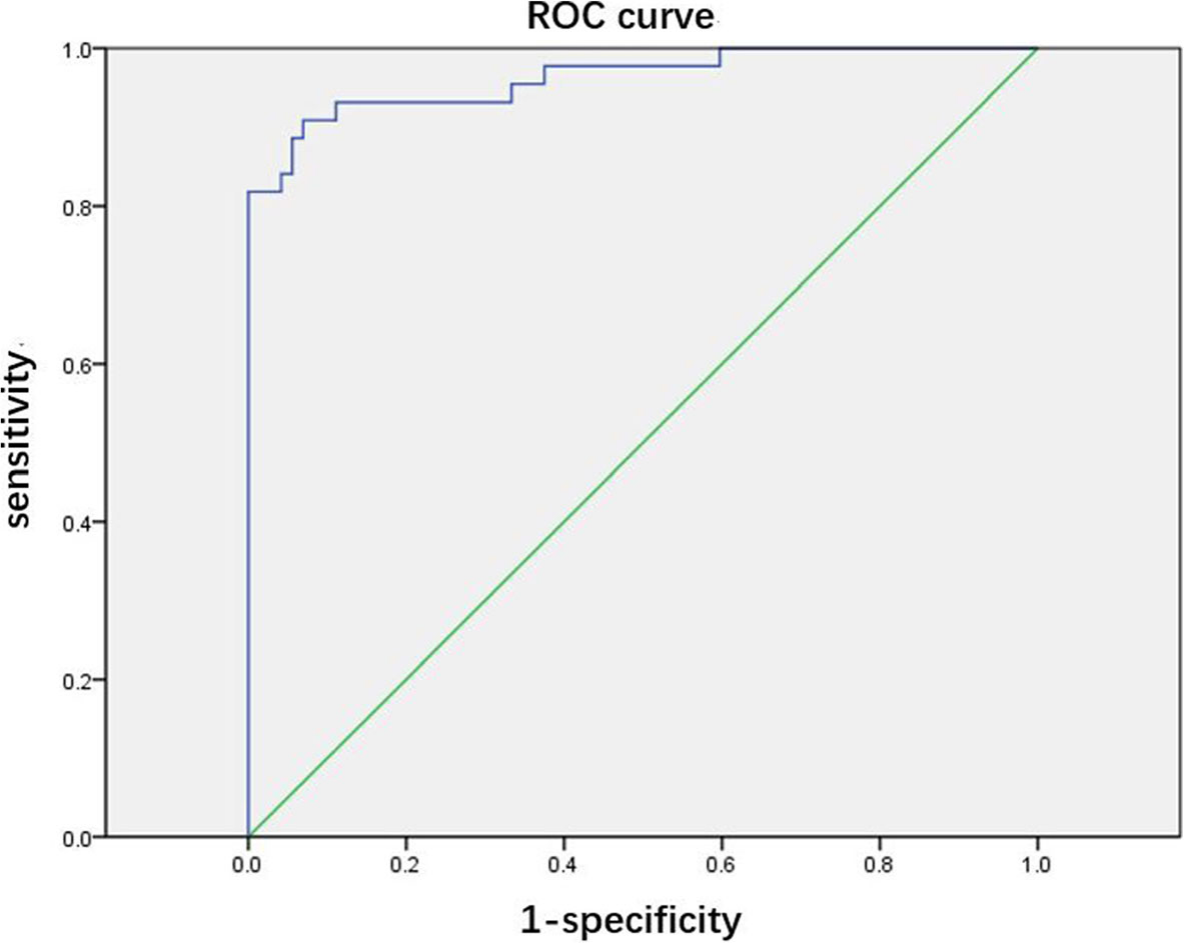
Receiver operating characteristic (ROC) curve for the LDH/ADA ratio in differentiating between TPE and PPE. An area-under-the-curve (AUC) value of 0.9663 was obtained using the pleural fluid LDH/ADA ratio as the indicator for TPE identification, and the sensitivity, specificity, positive likelihood ratio (PLR), and negative likelihood ratio (NLR) were, respectively, 93.62%, 93.06%, 13.48, and 0.068 at a cut-off level of 16.20

Clinical and laboratory findings in the patients with TPE and PPE are shown in Table 1. The TPE and PPE groups had median ages of 51.5 years (range,17–82 years) and 59.0 years (range, 26–93 years), respectively (*P* < .05), and the male sex percentages in the 2 groups were 68.1% and 75%, respectively. Univariate analysis revealed that in comparison with the PPE group, the TPE group had an increased pleural fluid protein level (*P* < .05), but a significantly lower pleural fluid LDH level (*P* < .001). In contrast, no significant differences were observed between the 2 groups in blood albumin and blood LDH levels, or in pleural fluid ADA levels (Table 1). However, the pleural fluid LDH/ADA ratio in the TPE group was significantly lower than in the PPE group, and the sensitivity, specificity, positive likelihood ratio (PLR, and negative likelihood ratio (NLR) for identifying TPE were, respectively, 93.62%, 93.06%, 13.48, and 0.068 at a cut-off level of 16.20 (Fig. 1).

Subgroup analysis showed no significant differences in pleural fluid LDH levels between patients with TPE and UPPE, or in pleural ADA levels between patients with TPE and CPPE. However, there were significant differences in pleural fluid LDH/ADA ratios between patients with TPE and each of the PPE subgroups (UPPE, CPPE, or empyema) [*P* < .0001 for each subgroup].

## Discussion

Currently, a definitive diagnosis of TPE is made on the basis of the following criteria: (1) a positive AFB smear or positive cultures for MTB in pleural fluid and pleural tissue; (2) chronic granulomatous inflammation in pleural tissue; and (3) a clinical response to anti-tuberculosis treatment [3, 5]. However, in most studies, an ADA level ≥ 40 U/L in a lymphocytic exudate obtained via thoracentesis has been the most widely accepted indicator for a diagnosis of TPE [16]. Although PPE can be confirmed by a pleural exudate in patients with bacterial pneumonia, lung abscesses or bronchiectasis, it is still difficult to make a differential diagnosis due to the variety of PPE subcategories (from UPPE to empyema) [10], as well as the absence of disease-specificity in pleural effusion biomarkers [17, 18]. Making a definitive diagnosis of UPPE is always challenging as such patients may or may not present with symptoms and signs of pneumonia. Therefore, histological examinations via pleural biopsy need to be employed for a definitive diagnosis of pleural effusions. In the present study, ultrasound-guided cutting-needle biopsy was used in combination with a standard pleural biopsy to diagnose pleural effusions in all patients [19].

Use of the ADA level in pleural fluid has demonstrated high sensitivity and specificity for the differential diagnosis of TPE [6, 20]. However, conflicting data were obtained by Zaricet et al. [21] who reported a poor specificity as low as 70.4% for the ADA level in diagnosing TPE, despite an acceptable sensitivity of 89.2%. Furthermore, although a higher ADA level in pleural fluid is considered to be associated with a greater chance of TPE, most patients with UPPE and CPPE have a pleural ADA level of around 40 U/L or below [10, 22], and an extremely high ADA level should raise a suspicion of empyema or lymphoma [10]. Although a similar pleural fluid ADA level was evident in patients with TPE, PPE and CPPE in the present study, a significantly lower pleural fluid ADA level was seen in patients with UPPE in comparison with those with TPE (*P* < .0001), and a significantly higher pleural fluid ADA level was seen in patients with empyema (*P* < .0001).

Pleural fluid LDH is a frequently used biomarker to differentiate CPPE from UPPE, and a very high and isolated pleural fluid LDH level might be of specific diagnostic significance, especially for empyema [22]. We found that the pleural fluid LDH level was significantly lower in patients with TPE than in those with PPE (*P* < .001), and an even greater difference was evident when patients with TPE were compared with the CPPE and empyema subgroups (*P* < .0001). However, there was no significant significance in the pleural fluid LDH level between patients with TPE and the UPPE subgroup (*P* = .291). Consequently, the results of our study are consistent with previous research [23] suggesting that use of ADA and LDH levels in pleural fluid for discriminating between TPE and PPE in clinical practice can be challenging.

In view of the limitations of using pleural fluid ADA and LDH levels alone as biomarkers for differentiating between TPE and PPE, we combined the 2 parameters in an attempt to develop a predictor of TPE with acceptable specificity and sensitivity. Our findings revealed a significantly lower pleural fluid LDH/ADA ratio in the TPE group compared with the PPE group (*P* < .0001) and this difference was also evident in comparison with the 3 PPE subgroups (UPPE, CPPE, and empyema) [*P* < .0001]. A pleural fluid LDH/ADA ratio < 16.20 was found to provide a sensitivity of 93.62%, a specificity of 93.06%, a PLR of 13.48, and a NLR of 0.068 for TPE identification, yielding an area-under-the-curve (AUC) of 0.9663. Therefore, it was concluded that a pleural fluid LDH/ADA ratio lower than 16.20 is highly predictive of TPE, and that 16.20 can be used as the cut-off value to discriminate between TPE and PPE. This finding could be helpful in early clinical decision-making for the management of these patients, as it could lead to a better prognosis and avoidance of potential adverse consequences.

ADA, an enzyme secreted by mononuclear cells, lymphocytes, neutrophils and red blood cells (RBCs) [10, 24], is categorized as ADA-1 and ADA-2. However, only total ADA is routinely measured in our clinical practice. ADA-2, which is mainly expressed in and released from mononuclear cells and macrophages, correlates with intracellular infection such as TPE, and high levels of ADA-1 are always present in empyema [25, 26]. LDH, as a ubiquitous cytoplasmatic enzyme in virtually all major organ systems, usually increases in a nonspecific manner in response to cell damage or cell death [27]. Consequently, an elevated pleural fluid LDH level in exudative pleural effusions (such as TPE and PPE), is indicative of lung or pleural tissue damage and endothelial injury [27]. Most patients with TPE show chronic granulomatous inflammation in pleural tissue, and infiltration of mononuclear cells and macrophages. However, in those with PPE, pleural tissues always demonstrate acute inflammation and infiltration of neutrophil cells, with a large number of pus cells. In the present study, the pleural fluid LDH/ADA ratio was significantly lower in patients with TPE compared with those with PPE (*P* < .0001) which may be due to differences in the pathological nature of the 2 conditions, since TPE is a chronic granulomatous inflammation characterized by infiltration of mononuclear cells and marcrophages while PPE is an acute inflammatory condition depending on its pathological course. A similar extent of pleural tissue damage in patients with TPE and UPPE was reflected in the absence of a significant difference in pleural fluid LDH levels between these groups in our study (*P* = .291), but the level of mononuclear cells and macrophages was higher in UPPE than in TPE. Among the PPE subgroups, the severity of pleural cell damage was least in UPPE and greatest in empyema, leading to increased LDH levels and corresponding increases in the LDH/ADA ratio in the 3 subgroups (Table 2). These results indicate that the LDH/ADA ratio may be a useful indicator of pleural inflammatory responses.

The main limitation of our study was that only blood and pleural fluid levels of LDH and ADA were analyzed, which was a consequence of the retrospective nature of the study. However, the pleural fluid LDH/ADA ratio has been shown to be superior to LDH or ADA alone as a parameter for differentiation between TPE and PPE. Another limitation was that the patients with TPE and PPE included in our study were not representative of those with pleural effusions caused by other conditions such as connective tissue diseases [28] or MPE [29], who may also have a high pleural fluid ADA or LDH levels. In addition, the sample size was small, and prospective studies are required to verify the study’s results.

## Conclusions

This study has provided evidence that the pleural fluid LDH/ADA ratio is a useful indicator to distinguish TPE from PPE. The LDH/ADA ratio may also reflect the nature of pleural inflammation and the response to inflammation. Consequently, it may be useful for the early clinical management of patients with pleural effusion.

## Additional file

Additional file 1: Excel: Pleural fluid results of LDHADA of the TPE and PPE patients. (XLS 26 kb)

## Abbreviations

ADA: adenosine deaminase
AFB: acid-fast bacillus
CPPE: complicated parapneumonic effusions
LDH: lactate dehydrogenase
MPE: malignant pleural effusion
MTB: *Mycobacterium tuberculosis*
NLR: negative likelihood ratio
PLR: positive likelihood ratio
PPE: parapneumonic pleural effusion
TB: tuberculosis
TPE: tuberculous pleural effusion
UPPE: uncomplicated parapneumonic effusion
